# Clinical pathways matter for multimodal deep learning in early Alzheimer’s disease detection

**DOI:** 10.1038/s41598-026-57861-z

**Published:** 2026-06-21

**Authors:** Yao Lu, Solveig Kristina Hammonds, Alvaro Fernandez-Quilez

**Affiliations:** 1 Department of Computer Science and Electrical Engineering, University of Stavanger, Kjell Arholms Hus 41, 4021 Stavanger, Norway; 2 Department of Computer Science, University of Putra Malaysia, Jalan Universiti 1, 43400 Selangor, Malaysia; 3 SMIL, Department of Radiology, Stavanger University Hospital, Gerd- Ragna Bloch Thorsens Gate 8, 4011 Stavanger, Norway; 4 Centre for Age-Related Medicine (SESAM), Stavanger University Hospital, Gerd-Ragna Bloch Thorsens gate 8, Stavanger, 4011 Norway

**Keywords:** Alzheimer’s disease, Multi-modal, MRI, AI, Deep learning, Clinical features, Biomarkers, Computational biology and bioinformatics, Neurology, Neuroscience

## Abstract

Identifying individuals at risk of Alzheimer’s disease (AD), particularly in the preclinical and early stages, remains challenging. Although deep learning approaches based on structural MRI show promise as a non-invasive biomarker, existing multimodal models require task-specific training and depend on biomarkers that are not routinely available in clinical practice. Here, we propose a zero-shot multimodal feature extraction framework based on SigLIP that combines structural MRI embeddings with text embeddings of routinely collected clinical variables for early AD risk stratification in individuals at preclinical or mild cognitive impairment (MCI) stages. We evaluated the approach in 416 individuals from the ADNI cohort (age: 72.73 ± 6.7). SigLIP was used without fine-tuning to extract MRI and clinical text embeddings, which were combined into multimodal representations for individual-level AD risk prediction within 4 years. We further compared the model performance in a single-visit and two-visit settings to assess the value of longitudinal information and framework scalability. In the 1-visit setting, combining MRI embeddings with MMSE, age, and sex achieved an AUC of 0.91 ± 0.02, showing higher performance than the CSF Aβ42-based model (AUC 0.73 ± 0.08) and MMSE-based model (AUC 0.85 ± 0.22). In the 2-visit setting, performance was maintained or improved, supporting the scalability of the approach to longitudinal data. These findings suggest that multimodal fusion of SigLIP-derived MRI features and routinely collected clinical variables may provide a practical and scalable strategy for early AD risk progression prediction without task-specific training.

**Supplementary Information:**

The online version contains supplementary material available at https://doi.org/10.1038/s41598-026-57861-z.

## Introduction

Alzheimer’s disease (AD) is a progressive neurodegenerative disorder that develops over many years, typically progressing from a preclinical phase to mild cognitive impairment (MCI) and dementia^1^. In developed countries, AD affects approximately 1 in 10 older adults aged 65 and above, and over a third of those aged 85 and older^2^. Although early detection could support timely intervention and improve trial stratification, it remains challenging because early markers are subtle and individual clinical trajectories are heterogeneous^3^.

In clinical practice, assessment is anchored in patient history, cognitive testing and functional evaluation^4^. Cognitive test instruments such as the Clinical Dementia Rating (CDR) and the Mini-Mental State Examination (MMSE) can support staging and trial enrolment but their sensitivity is limited when impairment is mild^5^. Structural MRI is often acquired as part of the routine evaluation of cognitive symptoms, primarily to exclude alternative causes of impairment such as vascular lesions^6^. Although MRI can also reveal patterns of neurodegeneration consistent with AD, these changes may be subtle at early stages, and their interpretation depends on expertise and acquisition factors^6,7^.

Deep learning (DL) has shown promise in extracting imaging features relevant to AD from MRI^3,8,9^, potentially capturing complex patterns beyond predefined regions or voxel-wise statistics^10–12^. Existing works leverage 3D convolutional networks or more recent transformer-based architectures to T1-weighted MRI for AD-versus-control classification and for predicting MCI-to-AD conversion^13,14^. However, performance is typically highest in established dementia and degrades in prodromal cohorts, where disease-related changes are subtle and heterogeneous across individuals^15^. This has motivated multimodal approaches that integrate imaging with demographic and clinical measures to approximate clinical decision-making^16,17^.

Despite their promise, multimodal models are highly sensitive to clinical-variable choice and collection context^18^. In practice, clinical measurements are not universally available or synchronously acquired. The collection timing can vary relative to imaging, missingness is systematic, and cohorts reflect referral patterns and diagnostic escalation^19^. Moreover, a substantial fraction of multimodal work incorporates specialized or invasive biomarkers such as PET imaging or cerebrospinal fluid (CSF)^20^. These may be costly, invasive, or not routinely available in standard care^21^. Therefore, failure in accounting for those factors can yield optimistic performance estimates while limiting clinical use and adoption^8^.

Here, we propose a multimodal DL framework that is grounded in the clinical pathway of AD assessment. In this context, clinical pathway refers to data-availability scenarios encountered during AD diagnostic assessment rather than to a biological disease trajectory^22^. The data combinations presented in the study were therefore selected to represent clinically plausible data-availability scenarios during AD diagnostic assessment, and based on routinely collected clinical information, structural MRI, and gold standard diagnostic biomarkers such as CSF Aβ42. We encode structured clinical information as compact text descriptors and use Sigmoid Loss for Language–Image Pre-training (SigLIP) to align these descriptors with MRI-derived embeddings^23^. We evaluate our approach against unimodal baselines and an invasive gold standard diagnostic biomarker of AD (CSF Aβ42), to quantify how much diagnostic signal can be recovered from routine clinical variables when clinical context is respected. We hypothesize that pathway-aware multimodal fusion improves early AD detection relative to MRI-only and routine-clinical-only models, achieving performance comparable to gold standard-based approaches using CSF Aβ42.

## Methods

### Participants and study design

The data used in this study were sourced from the Alzheimer’s Disease Neuroimaging Initiative (ADNI; *adni.loni.usc.edu*), a large, ongoing longitudinal study launched in 2003 to understand the progression of AD from normal aging to MCI and early AD^24^. Participants were recruited in-person from clinical centers across North America. The present study utilized data from ADNI-1, ADNI-GO, ADNI-2, and ADNI-3.

The ADNI database provides a comprehensive collection of multimodal data, including structural and functional imaging, CSF and blood biomarkers, cognitive test scores, genetic profiles, and detailed clinical and demographic information. In this study, these resources were utilized to evaluate the predictive value of clinical and imaging features for AD progression. The analyzed cohort comprised participants categorized as cognitively normal (CN), MCI or AD according to ADNI diagnostic protocols^24^.

To ensure consistency in imaging quality and reduce confounding from unrelated clinical factors, we applied the following inclusion criteria: (1) individuals with a baseline 1.5T or 3.0T T1-weighted MP-RAGE MRI scan and without dementia-unrelated neurological or psychiatric disorders; (2) a baseline clinical diagnosis of either MCI or CN; (3) at least two available visits; (4) available baseline clinical diagnosis, age, sex, APOE genotype, CDR and MMSE; (5) baseline MRI scans of diagnostic quality, as defined in^24^.

**Fig. 1:**
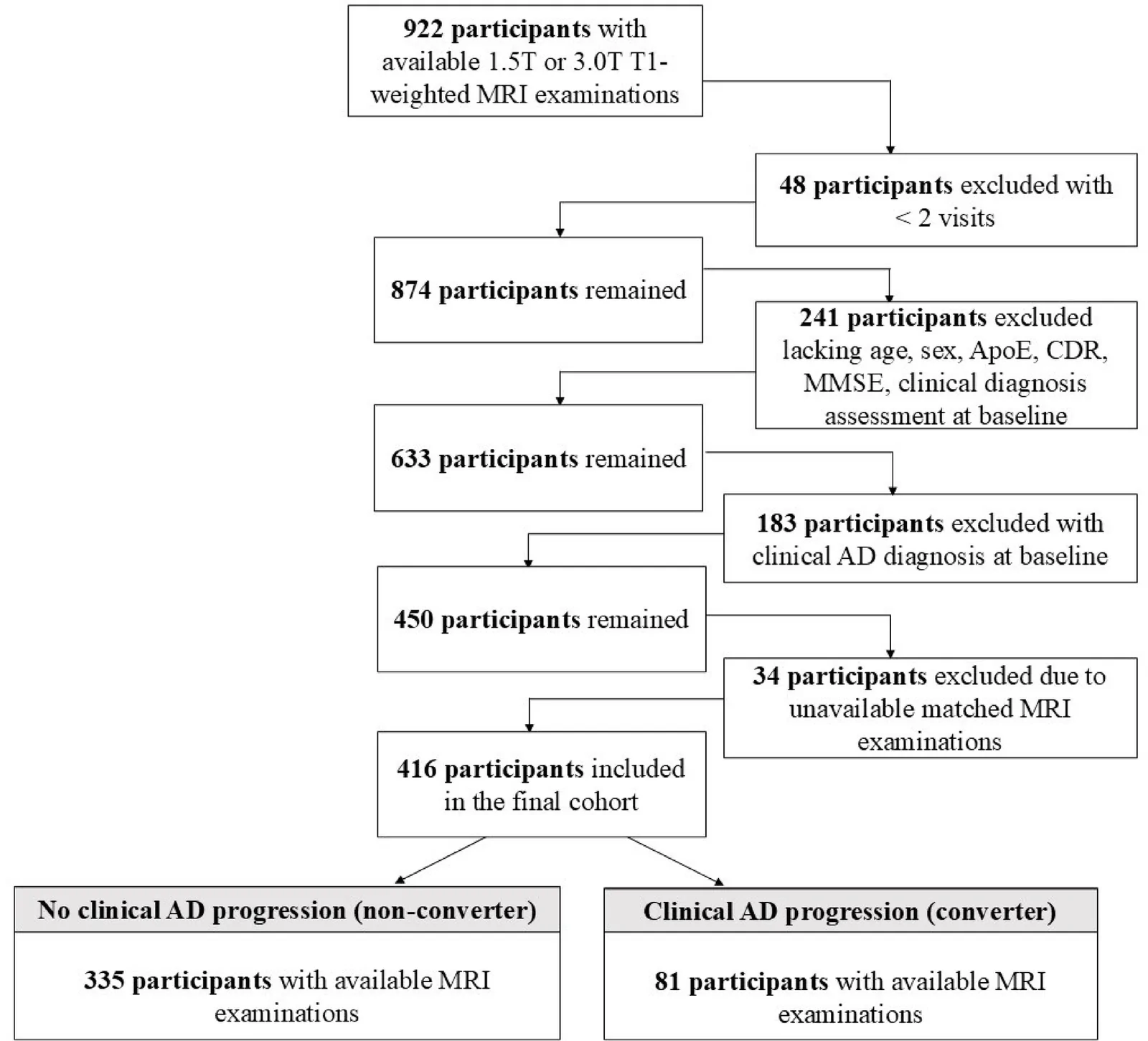
Flowchart of subject-level cohort selection. The screening process identified 416 eligible participants from an initial cohort of 922 participants. AD = Alzheimer’s disease, CDR = Clinical Dementia Rating, MMSE = Mini-Mental State Examination, APOE = Apolipoprotein E.

Eligible participants were divided into two groups based on clinical progression within a 4-year follow-up window from baseline: converters, who progressed from CN or MCI at baseline to a diagnosis of probable AD at any follow-up visit, and non-converters, defined as participants who retained a non-AD diagnosis throughout their available follow-up within the same window. The 4-year window was selected based on the overall follow-up distribution in the cohort and to provide a consistent time frame for defining progression status. Participants were required to have a minimum of 2 visits to assess progression. After applying these criteria, 416 unique participants met all inclusion criteria, including 81 converters and 335 non-converters. The subject-level selection process and resulting cohort are illustrated in Fig. 1.

### MRI and pre-processing

MRI scan-level filtering was subsequently performed within the cohort of 416 eligible participants (Fig. 1). A total of 2,718 candidate MRI examinations associated with these participants were initially identified and matched to the corresponding clinical visits. For each participant-visit pair, standard MP-RAGE acquisitions were retained, and identified repeat acquisitions were excluded. If multiple eligible scans remained after this filtering step, the earliest acquired scan of each participant was selected. This scan is thereby considered as baseline during the manuscript. This procedure resulted in 932 MRI scans in the final imaging dataset.

The retained T1-weighted MRI scans were acquired on either 1.5T or 3.0T scanner systems. The images were acquired across multiple scanner vendors between September 2002 and April 2022 using commercial scanners manufactured by Siemens Healthineers (Erlangen, Germany), Philips Medical Systems (Eindhoven, The Netherlands), and GE Healthcare (Chicago, Illinois, USA). ADNI applies standardized MRI protocols depending on field strengths, head coil and scanner type, with a nominal FOV of approx. 240 × 240 mm^2^, number of slices in the range of 160–208, and a voxel size of approx. 1 × 1 × 1 mm^3^^25^. ADNI also applies standardized acquisition protocols and scan-level quality control before MRI data release. Scans that did not meet ADNI image-quality requirements were not included in the final imaging dataset^26^.

**Fig. 2:**
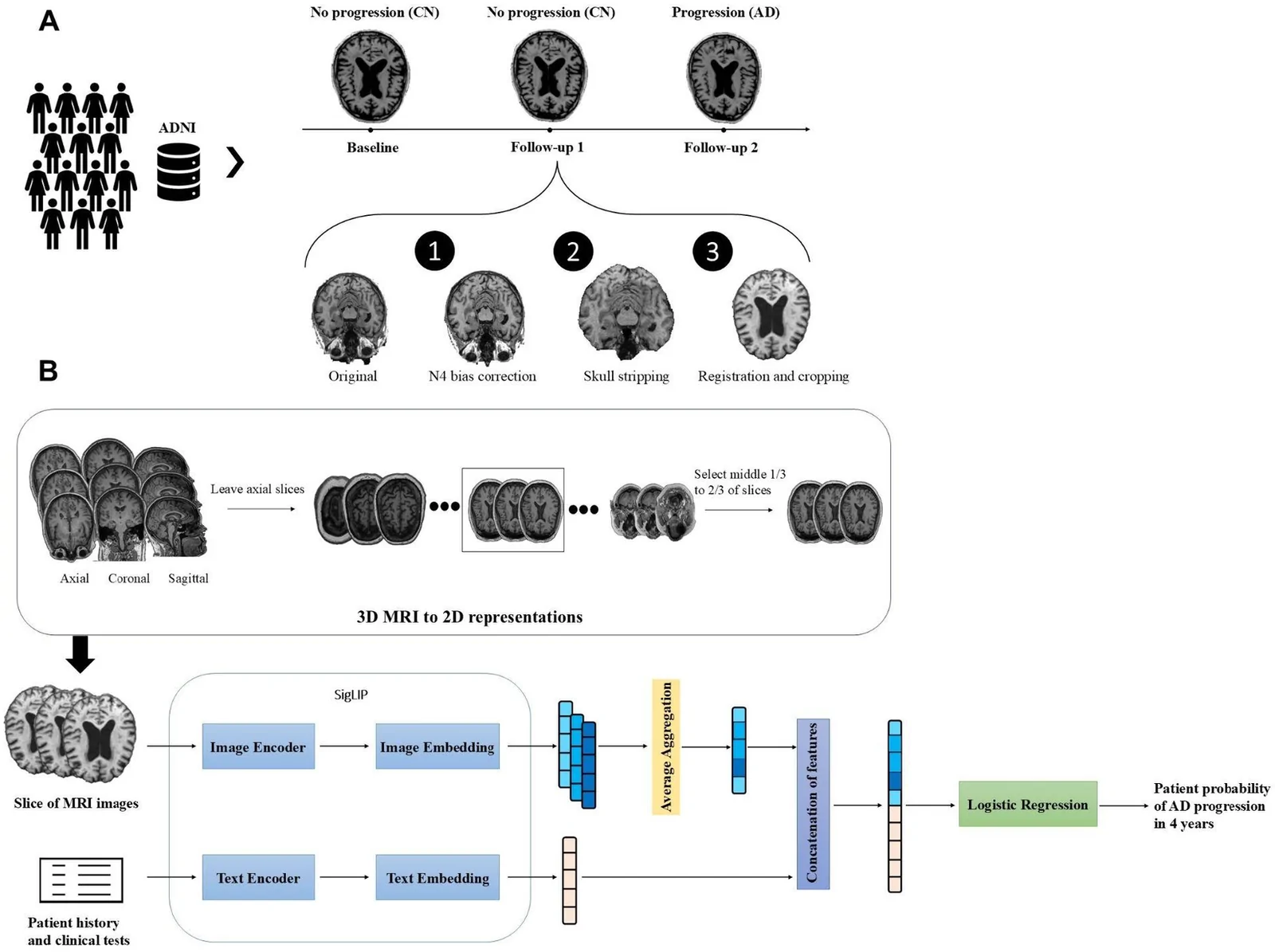
Method overview (A) ADNI dataset with longitudinal T1-weighted MRI data, including progression annotation and MRI pre-processing step (B) Multimodal classification pipeline using SigLIP to extract visual and textual embeddings from 2D T1-weighted MRI slices and clinical data, with concatenated features input into a logistic regression classifier for AD prediction. CN = Cognitively normal, AD = Alzheimer’s disease.

To reduce inter-subject and inter-scanner variability, all images were preprocessed using a Python-based pipeline adapted from the open-source ADNI preprocessing toolkit (*adni_preprocessing*). This toolkit implements a standardized ADNI-oriented structural MRI preprocessing workflow, with processing choices informed by the Clinica framework, an established standardized platform for clinical neuroimaging analysis^27^. In our study, the preprocessing steps included: (1) N4 bias field correction^28^ to address low-frequency intensity non-uniformities (2) skull stripping to remove non-brain tissue and facilitate inter-subject brain alignment (3) registration to the MNI152 T1-weighted template^29^ using a mutual information–based cost function and B-spline interpolation to preserve anatomical structures (4) cropping from the original field of view 208 × 240 × 256 mm³ to a standardized volume of 182 × 218 × 182 mm³, ensuring spatial consistency across participants. All images after registration contained at least 182 slices in the z-direction, ensuring that no anatomical information was lost. All pre-processing steps and resulting images are presented in Fig. 2a.

#### Transformation from 3D MRI to 2D representations

The original input data consisted of preprocessed 3D T1-weighted MRI volumes. Since the SigLIP vision encoder operates on 2D images, each 3D volume was transformed into a set of 2D axial slices, which served as intermediate inputs for visual feature extraction. This process is shown in Fig. 2B. For each subject-visit MRI volume, axial slices corresponding to the central portion of the volume^30^ were selected. This selection was informed by prior MRI-based AD studies that prioritized central MRI slices using either fixed slice indices falling within the 1/3 to 2/3 range or proportional retention of the middle third of slices^31,32^. This strategy was intended to focus on anatomically informative regions, including the hippocampus and medial temporal lobe, which are known to exhibit early structural changes in AD^2,33,34^.

The analytical data structure was hierarchical, spanning the subject, visit, MRI-volume, and slice levels. Each subject could contribute multiple clinical visits, and after scan-level filtering, each retained subject-visit pair was represented by one baseline 3D T1-weighted MP-RAGE MRI volume. Each 3D MRI baseline volume was then decomposed into 2D axial slices for feature extraction. Importantly, these slices were used only as intermediate units for feature extraction and were not treated as independent samples for model training, prediction, or evaluation.

### Multimodal deep learning-based analysis

We developed a multimodal DL model that utilizes pretrained SigLIP^23^ encoders used solely for feature extraction without further training to obtain visual and textual embeddings from 2D T1-weighted MRI slices and corresponding patient demographic information and neuropsychological tests. The SigLIP encoders were not fine-tuned on AD-specific data. Thus, for the remainder of the manuscript we refer to as “zero-shot” the process of feature extraction with pretrained encoders.

For each 3D MRI in this study, visual features were independently extracted from the 2D axial slices corresponding to the baseline MRI (Fig. 2B). Image features extracted from individual slices were then stacked along the slice dimension to form a feature matrix, where each row represents the feature vector of a single slice. This matrix was averaged along the slice dimension to obtain a single MRI-level imaging representation for each 3D MRI volume^33–35^. The result was a single MRI-level image feature vector for each subject-visit pair, integrating visual information from all selected slices of the corresponding 3D MRI volume (Fig. 2B). In both the 1-visit and 2-visit settings, final predictions were generated from subject-level multimodal representations.

Structured clinical and demographic variables (age, sex, MMSE and CDR score) in the ADNI database were tokenized using SigLIP’s built-in tokenizer^23^. After tokenization, the resulting text was passed into SigLIP’s text encoder, which produced a 512-dimensional embedding for each visit. The MRI-level image feature vector derived from the corresponding 3D MRI volume was then concatenated with the visit-level clinical text embedding to form a 1024-dimensional visit-level multimodal feature vector. In the 1-visit setting, only the baseline visit-level multimodal representation was used for subject-level classification. In the 2-visit setting, multimodal feature vectors from the same participant were concatenated in chronological order to form a 2048-dimensional subject-level representation before classification. The earlier visit representation was placed before the later visit representation, thereby preserving visit order in the model input. Visit intervals were not modeled as separate numerical inputs.

The combined features were subsequently used to train a logistic regression model^36^ to predict the probability of AD progression within 4 years (Fig. 2B). Logistic regression was selected as a clinically well-established and interpretable model, providing a standardized and simple predictive framework that allows the contribution of SigLIP-derived embeddings to be evaluated without confounding effects introduced by more complex classifiers. This design allowed performance differences to be interpreted primarily in relation to the extracted representations rather than variations in model complexity.

To examine the effect of additional longitudinal information, we controlled the number of visits per subject. Specifically, two settings were compared: one using only the baseline visit (1-visit setting), and another including up to 2 visits (2-visit setting). This allowed us to assess the influence and scalability of SigLiP when using longitudinal data in the 4-year AD progression risk estimation.

All model comparisons were based on an identical complete-case subset across the included variables, resulting in a final analysis sample of 267 subjects. Training and test sets were split at the subject level to avoid cross-contamination, with 80% of the subjects for training and 20% for testing. All associated visits, MRI volumes, selected 2D slices, clinical variables, and derived embeddings from the same participant were assigned to the same partition. This subject-level partitioning ensured that no data from the same individual appeared in both the training and test sets, thereby minimizing the risk of within-subject information leakage.

All experiments were implemented using Python (version 3.10), PyTorch (version 2.5.1), and the Hugging Face transformers library (version 4.52.1). The logistic regression classifier implemented with Scikit-learn (version 1.0.2), was trained using the Limited-memory Broyden–Fletcher–Goldfarb–Shanno (LBFGS) optimizer with a maximum of 500 iterations (max_iter = 500) to ensure convergence. All training and inference procedures were conducted on a Windows 11 workstation with an Intel Core i5-1340P CPU and 16GB RAM.

### Evaluation

#### AD progression risk

To evaluate the predictive performance of the models across different experimental conditions, we adopted balanced accuracy (BACC), area under the ROC curve (AUC), and F1 score. These metrics offer complementary perspectives on model behavior and support a more robust assessment when the available dataset is limited^37–39^. In addition, confusion matrices were reported at false positive rate (FPR) thresholds of 0.1 and 0.2 to compare model sensitivity under predefined false-positive levels. These thresholds correspond to approximately 90% and 80% specificity, respectively, allowing assessment of sensitivity–specificity trade-offs between a more conservative low-false-positive setting and a more permissive early detection-oriented setting^39^.

#### Additive effect of available amount of point-of-care information

We conducted a series of experiments based on image and text features extracted by the SigLIP model to systematically evaluate the impact of different point-of-care and commonly available clinical and demographic patient information. Specifically, we considered the following combinations of text features with SigLIP: (a) CDR and age, (b) CDR, age and sex (c) MMSE and age, and (d) MMSE, age and sex. In all cases, combinations were tested jointly with MRI. These combinations were selected to simulate a range of clinically meaningful scenarios with different amounts of available clinical information. To assess the incremental contribution of MRI embeddings, we additionally evaluated corresponding clinical-only models using the same clinical and demographic variables without MRI input. These results are reported in Appendix Tables S3 and S4 (Appendix Table S3 and Table S4). This experimental setup allows us to address how clinically asynchronous and different combinations of structured information affect 4-year risk estimation performance of AD.

#### Text-encoding strategies

For each feature combination, we evaluated three encoding strategies to convert structured variables into text inputs: (a) Text without value, (b) Value without text, and (c) a combined format including both Text and value (Table S1). This approach aimed to have a systematic understanding of how different encoding methods influence the model’s ability to capture meaningful patterns from clinical and demographic features encoded as text. Specifically, we assessed the individual contributions of each strategy to predictive performance and examined whether the text encoder could effectively utilize both contextual information and numerical patient data. As a representative case to depict the encoding process and experimentation with the variables, consider the variable CDR and the effect of different encoding strategies: (a) CDR value presented as plain numeric string “0.5”, directly presenting the severity rating without contextual information; (b) a semantic-only representation was used “Clinical Dementia Rating:“, which reflects a typical clinical phrase but omits the numeric value; (c) a hybrid format was adopted that combines both the numerical score and descriptive text, “Clinical Dementia Rating: 0.5”, to better resemble real-world clinical documentation (Appendix Table S5). For each feature combination, we maintained the same dataset splits and image features across different encoding strategies, ensuring that only the textual representation varied.

#### Baselines

To establish a baseline performance, we implemented logistic regression on both individual and combined clinical variables in the form of a numerical input and with no further encoding. This included: (a) CDR, (b) MMSE, (c) CSF Aβ42, (d) CSF Aβ42 with CDR and age, (e) CSF Aβ42 with CDR, age and sex, (f) CSF Aβ42 with MMSE and age, and (g) CSF Aβ42 with MMSE, age and sex. These baseline models reflect the relative accessibility of clinical features in practice: CDR, MMSE, age, and sex can be easily obtained through routine assessments and provide a reference for evaluating the potential benefits of multimodal integration with SigLIP. We leverage CSF Aβ42 as our main clinical reference standard, as that is typically the diagnostic gold standard for AD confirmation (Table 3)^4^.

### Statistical testing

Continuous variables that were normally distributed were summarized as mean ± standard deviation (SD). Categorical variables were reported as counts and percentages. Group comparisons were conducted using parametric or non-parametric tests as appropriate based on distributions.

To quantify performance variability, we used a stratified bootstrapping approach on the test set. For each model, we performed 1000 bootstrap iterations with resampling stratified by AD progression. All performance metrics were calculated for each resampled test subset, resulting in a distribution of 1000 scores per metric. Based on these bootstrap distributions, performance was reported as mean ± standard deviation in the tables and for ROC curve comparisons. The same bootstrap procedure was applied consistently across all models and visit settings.

Statistical comparisons between models were performed using permutation tests. For each comparison, model performance was evaluated on the same test subjects for both the tested model and the reference model. The observed performance difference was calculated, and a null distribution was generated by repeatedly permuting model assignments and recalculating the performance difference. After accounting for multiple model comparisons with Bonferroni correction, P values < 0.01 were considered statistically significant. All statistical analyses were conducted in Python 3.10.1 (https://www.python.org/downloads/) with SciPy (https://scipy.org/) library.

## Results

### Sample description

The demographic and clinical characteristics of all participants, as well as those of converters and non-converters are presented in Table 1. Our study included 416 individuals, with an average age of 72.73 ± 6.70 years. Female participants made up a slightly lower portion of the cohort (49.04%) compared to male participants (50.96%). At the baseline visit, the majority of subjects were categorized as CN (61.06%). Among the participants, 81 subjects were identified as converters, while the remaining were non-converters. On average, converters presented lower MMSE (26.41 ± 2.18) and higher CDR (0.50 ± 0.16) values than non-converters.

**Table 1:** Baseline demographic and clinical characteristics of all participants, along with data for converters and non-converters in the 4-year-follow up study. These characteristics were calculated separately for each group. This breakdown allows for a more detailed comparison of the baseline features between converters and non-converters. Non-converters: All records from individuals that are CN or MCI and never progress to AD. Converters: All records from individuals that are CN or MCI and progress to AD. APOE: Apolipoprotein E, CN: Cognitively normal, MCI: Mild Cognitive Impairment, CDR: Clinical Dementia Rating, MMSE: Mini-Mental State Examination. The p-values were computed for comparisons between non-converters and converters. p-values < 0.01 were considered statistically significant.

	All	Non-converters	Converters	Statistics
Participants				
Total,	416	335	81	
Male: Female	212:204	165:70	47:34	
Female %	49.04	50.75	41.98	*p* = 0.29
Baseline diagnosis				
CN: MCI	254:162	250:85	4:77	
MCI, %	38.94	25.37	95.06	*p* < 0.01
Age [years]	72.76 ± 6.70	72.59 ± 6.76	73.29 ± 6.46	*p* = 0.39
Cognitive tests				
MMSE	28.28 ± 1.93	28.74 ± 1.55	26.41 ± 2.18	*p* < 0.01
CDR	0.22 ± 0.26	0.16 ± 0.24	0.50 ± 0.16	*p* < 0.01
APOE genotype				
APOE4-,0,N	269	236	28	
APOE4 + 1/2, N	118/29	84/15	39/14	*p* < 0.01

**Fig. 3:**
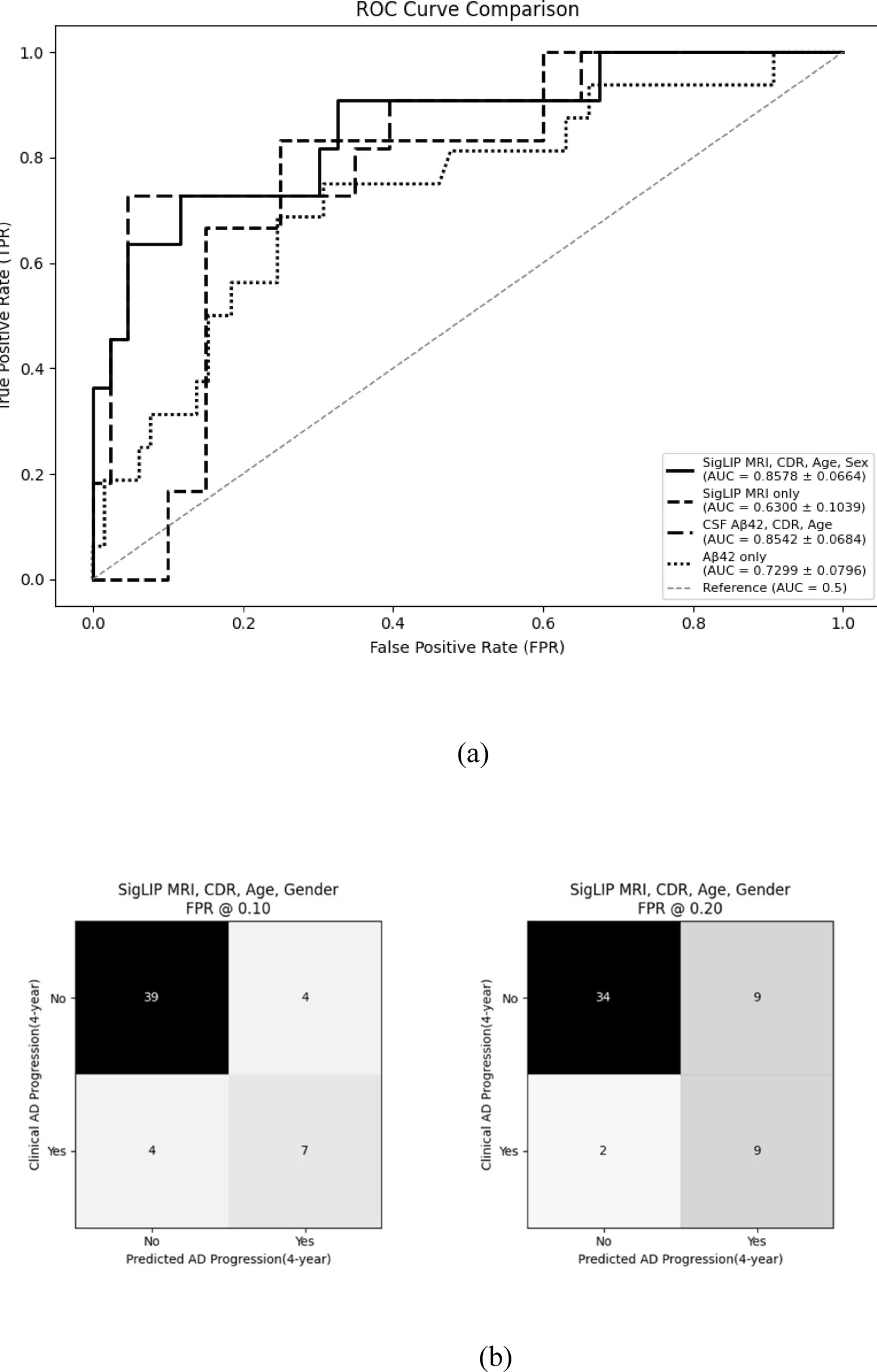
(**a**). Discriminative ability of four logistic regression models under the 1-visit setting, including unimodal models using only Aβ42 and only SigLIP MRI, and multimodal combinations of SigLIP MRI with CDR and age, and SigLIP MRI with CDR, age and sex. (**b**) Confusion matrices of the SigLIP-based multimodal model integrating image embeddings and clinical features (CDR, age, sex), under the 1-visit setting. The two matrices correspond to fixed FPR thresholds of 0.1 and 0.2 respectively, illustrating the trade-off between sensitivity and specificity at different decision thresholds. CDR = Clinical Dementia Rating. FPR= false positive rate.

Figure 3a depicts that multimodal combinations outperform unimodal approaches in the 1-visit setting, as further reflected in the confusion matrices calculated on the 20% test set of the final analysis sample (Fig. 3(b)). For the multimodal model with SigLIP MRI embeddings and clinical features (CDR, age, sex), confusion matrices at FPR thresholds of 0.1 and 0.2 were analyzed. At FPR = 0.1, the model identified 39 non-converters and 7 converters, with 4 false positives and 4 false negatives, indicating high specificity with moderate sensitivity. When the FPR threshold was relaxed to 0.2, false positives increased to 9, false negatives decreased to 2, and correctly predicted AD cases rose to 9, indication improve sensitivity with a moderate trade-off in specificity.

### Added value of multi-modal data

To investigate the effectiveness of multimodal information, we first focused on the model performance under the 1-visit setting (Table 3). SigLIP MRI alone did not perform as well as Aβ42 alone, but when combined with clinical features, such as CDR and age, SigLIP MRI showed a substantial improvement in performance, highlighting the complementary value of integrating imaging-derived features with clinical data. Among the multimodal combinations, the best performance was achieved by combining SigLIP MRI with MMSE, Age and Sex (AUC = 0.91 ± 0.02), which outperformed both Aβ42 alone (AUC = 0.73 ± 0.08) and SigLIP MRI alone (AUC = 0.63 ± 0.08).

### Effect of clinical features encoding

We conducted experiments with three types of encoding. Each encoding was integrated with Siglip-derived image features under both 1 visit and 2 visits. In the 1-visit condition (Table 2), numeric-only encoding performed best for SigLIP MRI with Age (AUC = 0.72 ± 0.18), while full-text encoding yielded the highest performance for SigLIP MRI with CDR and SigLIP MRI with CDR and age. In the 2-visit setting, full-text encoding outperformed all other methods (Appendix Table S1) see table 3.

**Table 2:** AUC performance of multimodal models using different text encodings with SigLIP MRI under the 1-visit setting. All experiments were conducted using the same dataset splits and MRI features to ensure a fair comparison. Results were computed using 5-fold cross-validation with bootstrapping (*N* = 1000) and are presented as mean ± standard deviation. CSF = cerebrospinal fluid; CDR = Clinical Dementia Rating; MMSE = Mini-Mental State Examination. * indicates the p-value for Text without value, calculated using Text and value as the reference; ** indicates the p-value for Value without text, calculated using Text and value as the reference. P-values < 0.01 were considered statistically significant.

Model	Text without value	Value without text	Text and value	Statistics
SigLIP MRI, Age AUC	0.55 ± 0.21	0.72 ± 0.18	0.70 ± 0.20	*p* < 0.01* *p* < 0.01**
SigLIP MRI, CDR AUC	0.63 ± 0.27	0.80 ± 0.08	0.84 ± 0.04	*p* < 0.01* *p* < 0.01**
SigLIP MRI CDR, Age AUC	0.65 ± 0.23	0.85 ± 0.07	0.90 ± 0.06	*p* < 0.01* *p* < 0.01**

**Table 3:** Classification performance of univariate and multimodal models under 1 visit. Values are calculated with stratified bootstrap resampling of *N* = 1000 repetitions and presented as mean ± standard deviation across bootstrap resamples. CSF = Cerebrospinal fluid, CDR = Clinical Dementia Rating, MMSE = Mini-Mental State Examination. P-value: Each comparison of the experimental group with the baseline reference group (SigLIP MRI, CDR, Age, Sex). P-values are calculated using permutation tests, with *p* < 0.01 indicating significant differences after correcting for multiple comparisons with Bonferroni correction.

Model	AUC	F1	Balanced accuracy	Statistics
Baseline CDR	0.84 ± 0.08	0.29 ± 0.26	0.59 ± 0.09	*p* = 0.35
Baseline MMSE	0.85 ± 0.22	0.45 ± 0.65	0.66 ± 0.29	*p* = 0.91
Invasive baseline				
CSF Aβ42	0.73 ± 0.08	0.27 ± 0.22	0.58 ± 0.08	*p* < 0.01
CSF Aβ42, CDR, Age	0.85 ± 0.07	0.40 ± 0.28	0.63 ± 0.12	*p* = 0.99
CSF Aβ42, CDR, Age, Sex	0.86 ± 0.11	0.53 ± 0.18	0.73 ± 0.13	*p* = 0.44
CSF Aβ42, MMSE, Age	0.84 ± 0.07	0.62 ± 0.11	0.77 ± 0.07	*p* = 0.94
CSF Aβ42, MMSE, Age, Sex	0.85 ± 0.08	0.62 ± 0.11	0.77 ± 0.07	*p* = 0.97
Non-invasive proposed				
SigLIP MRI	0.63 ± 0.08	0.45 ± 0.21	0.63 ± 0.11	*p* < 0.01
SigLIP MRI, CDR, Age	0.90 ± 0.06	0.75 ± 0.20	0.83 ± 0.15	*p* < 0.01
SigLIP MRI, CDR, Age, Sex	0.85 ± 0.07	0.52 ± 0.18	0.69 ± 0.10	Reference
SigLIP MRI, MMSE, Age	0.89 ± 0.02	0.74 ± 0.05	0.82 ± 0.03	*p* < 0.01
SigLIP MRI, MMSE, Age, Sex	0.91 ± 0.02	0.74 ± 0.05	0.82 ± 0.03	*p* < 0.01

### Effect of additional visits

**Fig. 4:**
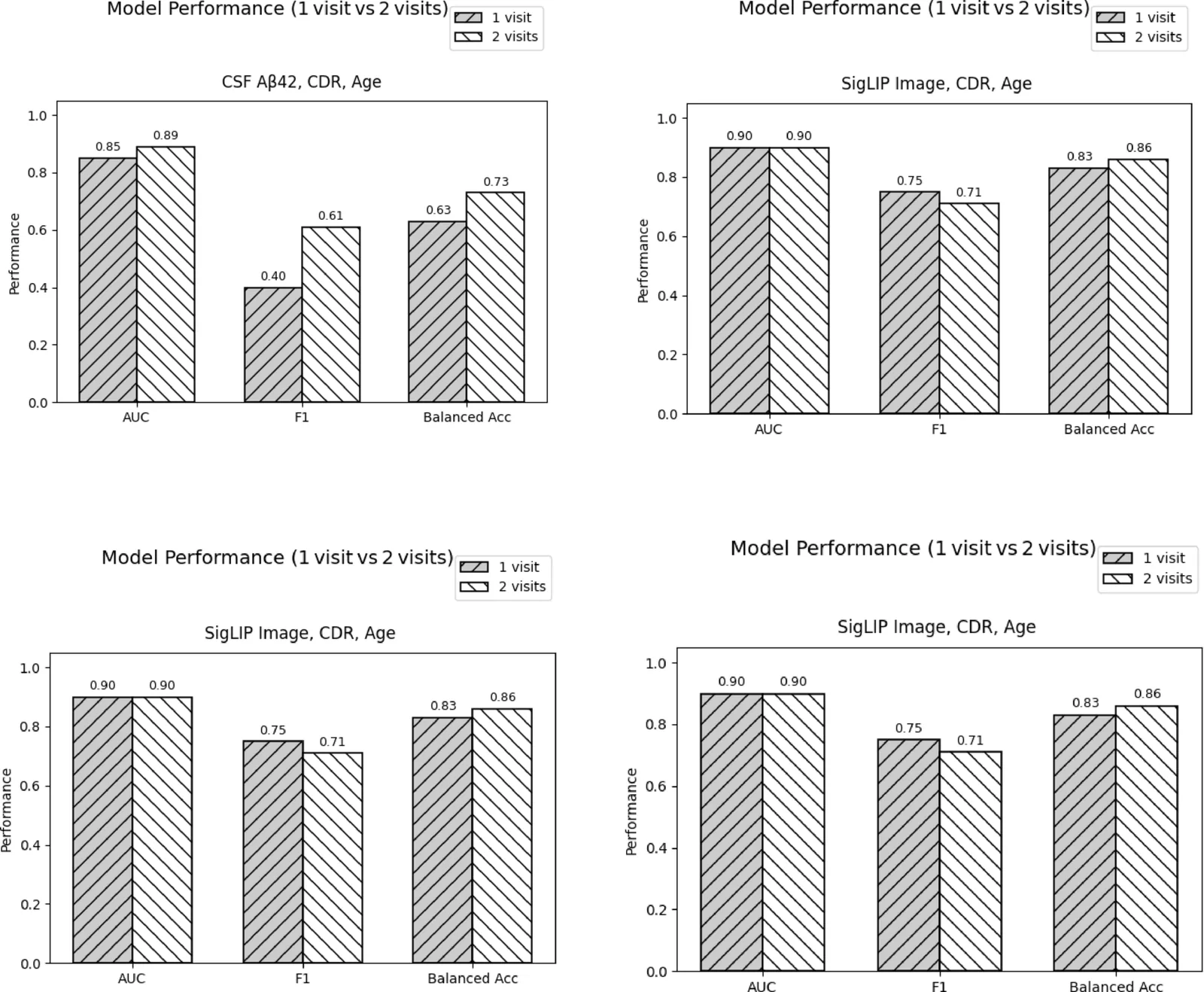
Comparison of model performance between 1-visit and 2-visit setting. The models cover a range of unimodal and multimodal configurations, including clinical variables (CDR and MMSE), combined clinical features (model with CSF Aβ42, CDR, age), and various multimodal combinations (SigLIP MRI with CDR, age, and sex). Performance is evaluated using AUC, F1 score, and balanced accuracy. CSF = Cerebrospinal fluid, CDR = Clinical Dementia Rating, MMSE = Mini-Mental State Examination.

As illustrated in Fig. 4, most models showed improved performance in the 2-visit setting compared with the 1-visit setting. The model of SigLIP MRI with CDR, age, and sex (AUC = 0.92 ± 0.03) achieved the highest performance in the 2-visit condition (Appendix Table S2). However, the performance of SigLIP MRI with MMSE and age, and SigLIP MRI with MMSE, age and sex was slightly lower in the 2-visit setting compared to the 1-visit condition.

## Discussion

In this retrospective study, we systematically evaluated SigLIP-based unimodal and multimodal classification models for predicting the 4-year risk of AD progression. Our findings demonstrate the feasibility of integrating MRI-derived features with routely collected clinical indicators through pretrained SigLIP encoders while accounting for variable clinical data availability.

Whereas many MRI-based deep learning approaches rely on task-specific training or fine-tuning for AD classification or progression prediction^40–43^, the proposed framework uses pretrained SigLIP encoders without AD-specific training, extending vision-language representations to zero-shot multimodal feature extraction for AD progression risk prediction. In contrast to prior work that has predominantly relied on invasive or costly biomarkers^44^, such as cerebrospinal fluid amyloid and tau measurements or FDG–PET imaging^45^, our study explores a more accessible strategy for multimodal AD progression prediction within a SigLIP framework. CSF Aβ42 was used as a benchmark comparator representing an established invasive biomarker. The results show that combining MRI-derived representations with clinical and demographic variables provided complementary predictive value, supporting the potential of a practical framework for non-invasive multimodal risk stratification across heterogeneous patient pathways.

This work also contributes methodologically by examining how clinical information is represented and how limited longitudinal information can be incorporated. Across most settings, text and value encoding performed better than representations based only on text without value or value without text, suggesting that preserving both qualitative and quantitative aspects of patient data may support more informative patient-level modeling^46–48^. Previous studies reduce clinical variables to structured numerical inputs^15,49^, which may fail to capture information contained in richer clinical descriptions. Unlike prior studies that relied on single-visit inputs^50–52^, we further compared the scalability of our method by comparing 1-visit and 2-visit settings. The benefit of adding a second visit was not uniform across feature combinations. For example, SigLIP MRI with CDR, age, and sex improved from an AUC of 0.85 in the 1-visit setting to 0.92 in the 2-visit setting, whereas SigLIP MRI with MMSE, age, and sex slightly decreased from 0.91 to 0.90. These findings suggest that additional useful information, including longitudinal data, can improve performance when it provides complementary signals beyond baseline clinical measures.

Our study had several limitations. Baseline diagnostic composition was imbalanced between converters and non-converters. Most converters had a baseline diagnosis of MCI, whereas most non-converters were CN. This imbalance may have contributed to model performance, as the models may partly capture baseline disease severity in addition to progression-specific risk. Relatedly, CDR and MMSE are established indicators of baseline cognitive and functional impairment and may therefore contribute substantially to model performance. Although comparisons with corresponding clinical-only models without MRI input showed that adding MRI embeddings generally improved AUC, the incremental value of imaging should be interpreted in the context of the prognostic information already captured by these clinical measures. Therefore, these results should be interpreted as evidence of potential complementarity between MRI-derived embeddings and routine clinical predictors. In addition, our study was restricted to 1-visit and 2-visit settings, and the modest sample size may constrain generalizability. One limitation is that ADNI is not fully representative of typical referral or real-world clinical populations, but it provides standardized and well-characterized imaging and clinical information for AD research. Although converting 3D MRI volumes into 2D axial slices enabled compatibility with the SigLIP image encoder, this design may not fully preserve volumetric anatomical context. Moreover, averaging slice-level embeddings into a single MRI-level representation may dilute spatially localized AD-related neurodegenerative patterns. Future work should explore 3D-aware feature extraction or attention-based aggregation strategies that better preserve volumetric and localized anatomical information while retaining the advantages of pretrained vision-language representations. In addition, SigLIP encoders were used without AD-specific fine-tuning, and task-adapted optimization may further improve performance.

## Conclusion

Our study shows that structural MRI features extracted using SigLIP when combined with routinely available clinical biomarkers have potential as a non-invasive approach for predicting individual AD progression, whilst accounting for common AD clinical pathways. By leveraging zero-shot image feature extraction from a pretrained model, the framework showed feasibility for early risk estimation in both single-visit and longitudinal settings within a retrospective ADNI cohort. These findings support a potentially scalable and accessible approach for future AD progression prediction.

## Supplementary Information

Below is the link to the electronic supplementary material.

Supplementary Material 1

## Data Availability

The data used in this study were obtained from the Alzheimer’s Disease Neuroimaging Initiative (ADNI) database (http://adni.loni.usc.edu/), which is publicly available to qualified researchers upon application.

## References

[1] Chen, Y. et al. Progress on early diagnosing Alzheimer’s disease. *Front. Med.* **18**, 446–464. 10.1007/s11684-023-1047-1 (2024). 38769282 PMC11391414

[2] Twarowski, B. & Herbet, M. Inflammatory processes in Alzheimer’s disease-pathomechanism, diagnosis and treatment: A review. *Int. J. Mol. Sci.* **24**, 6518. 10.3390/ijms24076518 (2023). 37047492 PMC10095343

[3] Singh, S. G., Das, D., Barman, U. & Saikia, M. J. Early Alzheimer’s disease detection: A review of machine learning techniques for forecasting transition from mild cognitive impairment. *Diagnostics (Basel)* **14**, 1759. 10.3390/diagnostics14161759 (2024). 39202248 PMC11353639

[4] Jack, C. R. Jr et al. Revised criteria for diagnosis and staging of Alzheimer’s disease: Alzheimer’s Association workgroup. *Alzheimers Dement.* **20**, 5143–5169. 10.1002/alz.13859 (2024). 38934362 PMC11350039

[5] Liu, E., Zhang, Y. & Wang, J. Z. Updates in Alzheimer’s disease: From basic research to diagnosis and therapies. *Transl. Neurodegener.* **13**, 45. 10.1186/s40035-024-00432-x (2024). 39232848 PMC11373277

[6] Pérez-Millan, A. et al. Beyond group classification: probabilistic differential diagnosis of frontotemporal dementia and Alzheimer’s disease with MRI and CSF biomarkers. *Neurobiol. Aging*. **144**, 1–11. 10.1016/j.neurobiolaging.2024.08.008 (2024). 39232438

[7] Shanmugavadivel, K., Sathishkumar, V. E., Cho, J. & Subramanian, M. Advancements in computer-assisted diagnosis of Alzheimer’s disease: A comprehensive survey of neuroimaging methods and AI techniques for early detection. *Ageing Res. Rev.* **91**, 102072. 10.1016/j.arr.2023.102072 (2023). 37709055

[8] Vrahatis, A. G. et al. Revolutionizing the early detection of Alzheimer’s disease through non-invasive biomarkers: The role of artificial intelligence and deep learning. *Sensors (Basel)* **23**, 4184. 10.3390/s23094184 (2023). 37177386 PMC10180573

[9] Mora-Rubio, A. et al. Classification of Alzheimer’s disease stages from magnetic resonance images using deep learning. *PeerJ Comput. Sci.* **9**, e1490. 10.7717/peerj-cs.1490 (2023). 37705614 PMC10495979

[10] Malik, I., Iqbal, A., Gu, Y. H. & Al-Antari, M. A. Deep learning for Alzheimer’s disease prediction: A comprehensive review. *Diagnostics (Basel)* **14**, 1281. 10.3390/diagnostics14121281 (2024). 38928696 PMC11202897

[11] Park, C. J. et al. Deep learning-based quantification of brain atrophy using 2D T1-weighted MRI for Alzheimer’s disease classification. *Front. Aging Neurosci.* **16**, 1423515. 10.3389/fnagi.2024.1423515 (2024). 39206118 PMC11349618

[12] Fathi, S., Ahmadi, A., Dehnad, A., Almasi-Dooghaee, M. & Sadegh, M. A deep learning-based ensemble method for early diagnosis of Alzheimer’s disease using MRI images. *Neuroinformatics* **22**, 89–105. 10.1007/s12021-023-09646-2 (2024). 38042764 PMC10917836

[13] Huang, H. et al. Voxel-based morphometry and a deep learning model for the diagnosis of early Alzheimer’s disease based on cerebral gray matter changes. *Cereb. Cortex*. **33**, 754–763. 10.1093/cercor/bhac099 (2023). 35301516 PMC9890469

[14] Reddy, K. K. AlzheimerViT: harnessing lightweight vision transformer architecture for proactive Alzheimer’s screening. *Front. Med. (Lausanne)*. **12**, 1568312. 10.3389/fmed.2025.1568312 (2025). 40600046 PMC12210019

[15] Wang, Y. et al. Predicting long-term progression of Alzheimer’s disease using a multimodal deep learning model incorporating interaction effects. *J. Transl Med.* **22**, 265. 10.1186/s12967-024-05025-w (2024). 38468358 PMC10926590

[16] Chen, Y. et al. Toward robust early detection of Alzheimer’s disease via an integrated multimodal learning approach. In: *ICASSP 2025 IEEE International Conference on Acoustics, Speech and Signal Processing* (IEEE), 1–5 (2025).

[17] Venugopalan, J., Tong, L., Hassanzadeh, H. R. & Wang, M. D. Multimodal deep learning models for early detection of Alzheimer’s disease stage. *Sci. Rep.* **11**, 3254. 10.1038/s41598-020-74399-w (2021). 33547343 PMC7864942

[18] Ai, M. et al. Research progress in predicting the conversion from mild cognitive impairment to Alzheimer’s disease via multimodal MRI and artificial intelligence. *Front. Neurol.* **16**, 1596632 (2025). 40529431 10.3389/fneur.2025.1596632 PMC12171368

[19] Azad, R. et al. Addressing missing modality challenges in MRI images: a comprehensive review. *Computational Visual Media* **11**, 241–268 (2025).

[20] Kim, S. K., Duong, Q. A. & Gahm, J. K. Multimodal 3D deep learning for early diagnosis of Alzheimer’s disease. *IEEE Access* (2024).

[21] Odusami, M., Maskeliūnas, R., Damaševičius, R. & Misra, S. Explainable deep-learning-based diagnosis of Alzheimer’s disease using multimodal input fusion of PET and MRI images. *J. Med. Biol. Eng.* **43**, 291–302 (2023).

[22] Hampel, H. et al. Designing the next-generation clinical care pathway for Alzheimer’s disease. *Nat. Aging*. **2**, 692–703. 10.1038/s43587-022-00269-x (2022). 37118137 PMC10148953

[23] Tschannen, M. et al. SigLIP 2: multilingual vision-language encoders with improved semantic understanding, localization, and dense features. Preprint at (2025). https://arxiv.org/abs/2502.14786

[24] Jack, C. R. Jr et al. The Alzheimer’s Disease Neuroimaging Initiative (ADNI): MRI methods. *J. Magn. Reson. Imaging*. **27**, 685–691. 10.1002/jmri.21049 (2008). 18302232 PMC2544629

[25] Arani, A. et al. Design and validation of the ADNI MR protocol. *Alzheimers Dement.* **20**, 6615–6621. 10.1002/alz.14162 (2024). 39115941 PMC11497751

[26] Jack, C. R. Jr. et al. Alzheimer’s Disease Neuroimaging Initiative. Overview of ADNI MRI. *Alzheimers Dement.* **20**, 7350–7360. 10.1002/alz.14166 (2024). 39258539 PMC11485416

[27] Routier, A. et al. Clinica: An open-source software platform for reproducible clinical neuroscience studies. *Front. Neuroinform*. **15**, 689675. 10.3389/fninf.2021.689675 (2021). 34483871 PMC8415107

[28] Tustison, N. J. et al. N4ITK: improved N3 bias correction. *IEEE Trans. Med. Imaging*. **29**, 1310–1320. 10.1109/TMI.2010.2046908 (2010). 20378467 PMC3071855

[29] Fonov, V. S. et al. Unbiased nonlinear average age-appropriate brain templates from birth to adulthood. *NeuroImage* **47**, S102. 10.1016/S1053-8119(09)70884-5 (2009).

[30] Kumar, S. V., Supraja, B. S., Maheshwari, B. N. & Madhuri, B. Memory loss and Alzheimer’s disease progression convolutional neural networks with dropout layers for optimal filtered features in MRI images of the hippocampus for slice selection based on landmarks. *Turk. J. Comput. Math. Educ.* **15**, 154–163 (2024).

[31] Şener, B., Açıcı, K. & Sümer, E. Improving early detection of Alzheimer’s disease through MRI slice selection and deep learning techniques. *Sci. Rep.* **15**, 29260. 10.1038/s41598-025-14476-0 (2025). 40784967 PMC12336342

[32] Stoleru, G. I. & Iftene, A. Transfer learning for Alzheimer’s disease diagnosis from MRI slices: A comparative study of deep learning models. *Procedia Comput. Sci.* **225**, 2614–2623. 10.1016/j.procs.2023.10.253 (2023).

[33] Rao, G. et al. MRI measurements of brain hippocampus volume in relation to mild cognitive impairment and Alzheimer disease: a systematic review and meta-analysis. *Med. (Baltim)*. (2023). 10.1097/MD.0000000000034997 (2023). PMC10489245 37682140

[34] Pusparani, Y. et al. Hippocampal volume asymmetry in Alzheimer disease: a systematic review and meta-analysis. *Med. (Baltim).* **104**, e41662. 10.1097/MD.0000000000041662 (2025). PMC11903023 40068028

[35] Yaniv, Z., Lowekamp, B. C., Johnson, H. J. & Beare, R. SimpleITK image-analysis notebooks: a collaborative environment for education and reproducible research. *J. Digit. Imaging*. **31**, 290–303. 10.1007/s10278-017-0037-8 (2018). 29181613 PMC5959828

[36] Schober, P. & Vetter, T. R. Logistic regression in medical research. *Anesth. Analg*. **132**, 365–366. 10.1213/ANE.0000000000005247 (2021). 33449558 PMC7785709

[37] Gade, M. et al. Impact of uncertainty quantification through conformal prediction on volume assessment from deep learning-based MRI prostate segmentation. *Insights Imaging*. **15**, 286. 10.1186/s13244-024-01863-w (2024). 39613981 PMC11607187

[38] Anaya-Isaza, A., Mera-Jiménez, L. & Fernandez-Quilez, A. CrossTransUnet: a new computationally inexpensive tumor segmentation model for brain MRI. *IEEE Access.* **11**, 27066–27078. 10.1109/ACCESS.2023.3257767 (2023).

[39] Hammonds, S. K. et al. Decision strategies in AI-based ensemble models in opportunistic Alzheimer’s detection from structural MRI. *J. Digit. Imaging*. 10.1007/s10278-025-01604-5 (2025). PMC13230358 40963032

[40] Wang, J., Wen, S., Liu, W., Meng, X. & Jiao, Z. Deep joint learning diagnosis of Alzheimer’s disease based on multimodal feature fusion. *J. Ex. Stud.* **17**, 48. 10.1186/s13040-024-00395-9 (2024). PMC11536794 39501294

[41] Hammonds, S. K., Eftestøl, T., Oppedal, K. & Fernandez-Quilez, A. In: *4th International Conference on Applied Artificial Intelligence (ICAPAI)* (IEEE), 1–7. 10.1109/ICAPAI61893.2024.10541140 (2024).

[42] Mandal, P. K. & Mahto, R. V. Deep multi-branch CNN architecture for early Alzheimer’s detection from brain MRIs. *Sens. (Basel)*. **23**, 8192. 10.3390/s23198192 (2023). PMC10574860 37837027

[43] Slimi, H., Balti, A., Abid, S. & Sayadi, M. A combinatorial deep learning method for Alzheimer’s disease classification-based merging pretrained networks. *Front. Comput. Neurosci.* **18**, 1444019. 10.3389/fncom.2024.1444019 (2024). 39483205 PMC11525984

[44] Karlawish, J. & Grill, J. D. Alzheimer’s disease biomarkers and the tyranny of treatment. *EBioMedicine* **108**, 105291. (2024). 10.1016/j.ebiom.2024.105291 (2024). 39366841 PMC11663781

[45] Liu, E., Zhang, Y. & Wang, J. Z. Updates in Alzheimer’s disease: from basic research to diagnosis and therapies. *Transl Neurodegener*. **13**, 45. 10.1186/s40035-024-00432-x (2024). 39232848 PMC11373277

[46] Seinen, T. M. et al. Use of unstructured text in prognostic clinical prediction models: a systematic review. *J. Am. Med. Inf. Assoc.* **29**, 1292–1302. 10.1093/jamia/ocac058 (2022). PMC9196702 35475536

[47] Lu, Y., Lindeijer, T. N., Ytredal, T. M., Alvestad, A. B. & Fernandez-Quilez, A. AI-based prostate volume estimation from multi-planar MRI under variable acquisition protocols. *Eur. J. Radiol. Open.* **16**, 100738. 10.1016/j.ejro.2026.100738 (2026). 41783490 PMC12954287

[48] Sortland Rolfsnes, E. et al. On Undesired Emergent Behaviors in Compound Prostate Cancer Detection Systems. In *MICCAI Workshop on Cancer Prevention through Early Detection* 73–82 (Springer, 2024). 10.1007/978-3-031-73376-5_7.

[49] Henríquez, P. A. & Araya, N. Multimodal Alzheimer’s disease classification through ensemble deep random vector functional link neural network. *PeerJ Comput. Sci.* **10**, e2590. 10.7717/peerj-cs.2590 (2024). 39896355 PMC11784893

[50] Devanarayan, V. et al. Multimodal prognostic modeling of individual cognitive trajectories to enhance trial efficiency in preclinical Alzheimer’s disease. *Alzheimers Dement.* **21**, e70702. 10.1002/alz.70702 (2025). 40990131 PMC12457986

[51] Zarei, S., Saffar, M., Shalbaf, R., Hassani Abharian, P. & Shalbaf, A. Explainable hierarchical machine-learning approaches for multimodal prediction of conversion from mild cognitive impairment to Alzheimer’s disease. *Phys. Eng. Sci. Med.* **48**, 1741–1759. 10.1007/s13246-025-01618-x (2025). 40788534

[52] Hjertager Osenbroch, S., Ramona Rosvold, L., Lu, Y. & Fernandez-Quilez, A. Neuropsychiatric Deviations From Normative Profiles: An MRI-Derived Marker for Early Alzheimer’s Disease Detection. arXiv e-prints, arXiv-2604 (2026). 10.48550/arXiv.2604.00545

