# Supplementary material for: Clinical pathways matter for multimodal deep learning in early Alzheimer’s disease detection
Source: Sci Rep. 2026 Jun 21;16:28381. doi: 10.1038/s41598-026-57861-z (PMC13562722; doi:10.1038/s41598-026-57861-z)
Supplement: Supplementary file 1 [file 41598_2026_57861_MOESM1_ESM.pdf]

Supplementary Information

Title: Clinical Pathways Matter for Multimodal Deep Learning in Early Alzheimer's Disease Detection

Authors: Yao Lu[1,2], Solveig Kristina Hammonds[1,3,4] and Alvaro Fernandez-Quilez[1,*], for the Alzheimer's Disease Neuroimaging Initiative

Affiliations:

[1]Department of Computer Science and Electrical Engineering, University of Stavanger, Kjell Arholms Hus 41, 4021, Stavanger, Norway.
[2]Department of Computer Science, University of Putra Malaysia, Jalan Universiti 1, 43400, Selangor, Malaysia.
[3]SMIL, Department of Radiology, Stavanger University Hospital, Gerd-Ragna Bloch Thorsens Gate 8, 4011, Stavanger, Norway.
[4]Centre for Age-Related Medicine (SESAM), Stavanger University Hospital, Gerd-Ragna Bloch Thorsens gate 8, Stavanger, 4011, Norway
[*]Corresponding author: Alvaro Fernandez-Quilez, Department of Computer Science and Electrical Engineering, University of Stavanger, Kjell Arholms Hus 41, 4021, Stavanger, Norway. E-mail: alvaro.f.quilez@uis.no

	Text without value	Value without text	Text and value	Statistics
**SigLIP MRI, Age**<br>AUC	0.53 ± 0.34	0.71 ± 0.27	0.86 ± 0.18	p<0.01*<br>p<0.01**
**SigLIP MRI, CDR**<br>AUC	0.53 ± 0.44	0.82 ± 0.08	0.88 ± 0.20	p<0.01*<br>p<0.01**
**SigLIP MRI CDR, Age**<br>AUC	0.55 ± 0.35	0.86 ± 0.25	0.90 ± 0.04	p<0.01*<br>p<0.01**

Table S1. AUC Performance of multimodal models using different text encodings with SigLIP MRI under the 2-visit setting. All experiments were conducted on the same dataset splits and image

features to ensure fair comparison. Results are computed using 5-fold cross-validation, with bootstrapping (N = 1000) and presented as mean ± SD. CSF = Cerebrospinal fluid, CDR = Clinical Dementia Rating. * indicates the p-value for Text without value compared with Text and value as the reference. ** indicates the p-value for Value without text compared with Text and value as the reference. p-values < 0.01 were considered statistically significant.

Model	AUC	F1	Balanced accuracy	Statistics
Baseline CDR	0.87 ± 0.05	0.17 ± 0.16	0.55 ± 0.04	p<0.001
Baseline MMSE	0.90 ± 0.20	0.81 ± 0.43	0.84 ± 0.29	p=0.33
**Invasive baseline**				
CSF Aβ42	0.80 ± 0.10	0.31 ± 0.20	0.60 ± 0.07	p<0.001
CSF Aβ42, CDR, Age	0.89 ± 0.09	0.61 ± 0.23	0.73 ± 0.13	p=0.002
CSF Aβ42, CDR, Age, Sex	0.86 ± 0.10	0.66 ± 0.19	0.81 ± 0.14	p<0.001
CSF Aβ42, MMSE, Age	0.90 ± 0.04	0.64 ± 0.11	0.80 ± 0.06	p<0.001
CSF Aβ42, MMSE, Age, Sex	0.89 ± 0.04	0.60 ± 0.11	0.78 ± 0.06	p<0.001
**Non-invasive proposed**				
SigLIP MRI	0.57 ± 0.21	0.34 ± 0.30	0.60 ± 0.13	p<0.001
SigLIP MRI, CDR, Age	0.90 ± 0.04	0.71 ± 0.11	0.86 ± 0.06	p<0.001
SigLIP MRI, CDR, Age, Sex	0.92 ± 0.03	0.63 ± 0.10	0.83 ± 0.04	Reference
SigLIP MRI, MMSE, Age	0.90 ± 0.02	0.72 ± 0.04	0.81 ± 0.03	p<0.001
SigLIP MRI, MMSE, Age, Sex	0.90 ± 0.02	0.72 ± 0.04	0.81 ± 0.03	p<0.001

Table S2. Classification performance of univariate and multimodal models under 2 visits. Values are calculated with a bootstrap of N = 1000 repetitions and presented as mean ± standard deviation. The

probability threshold was optimized with respect to balanced accuracy for every case. CSF = Cerebrospinal fluid, CDR = Clinical Dementia Rating, MMSE = Mini-Mental State Examination. P-value: Each comparison of the experimental group with the baseline reference group (SigLIP MRI, CDR, Age, Sex). p-values calculated using independent t-tests, with $p < 0.001$ indicating highly significant differences.

Model	AUC	F1	Balanced accuracy	Statistics
SigLIP with CDR, Age	0.79 ± 0.05	0.42 ± 0.10	0.65 ± 0.06	Reference
SigLIP with CDR, Age, Sex	0.80 ± 0.05	0.60 ± 0.11	0.77 ± 0.06	p=0.014
SigLIP with MMSE, Age	0.70 ± 0.07	0.39 ± 0.09	0.62 ± 0.06	p<0.001
SigLIP with MMSE, Age, Sex	0.76 ± 0.05	0.45 ± 0.08	0.67 ± 0.05	p<0.001

Table S3. Classification performance of univariate and multivariable clinical models under 1 visit. Values are calculated using bootstrap resampling with N = 1000 repetitions and are presented as mean ± standard deviation. The probability threshold was optimized with respect to balanced accuracy for each model. CDR = Clinical Dementia Rating; MMSE = Mini-Mental State Examination. P-values indicate comparisons between each model and the reference SigLIP with CDR and Age, calculated using independent t-tests, with $p < 0.001$ indicating highly significant differences.

Model	AUC	F1	Balanced accuracy	Statistics
SigLIP with CDR, Age	0.79 ± 0.08	0.78 ± 0.12	0.61 ± 0.06	Reference
SigLIP with CDR, Age, Sex	0.81 ± 0.06	0.62 ± 0.10	0.75 ± 0.06	p<0.001
SigLIP with MMSE, Age	0.74 ± 0.06	0.57 ± 0.09	0.62 ± 0.04	p<0.001

SigLIP with MMSE, Age, Sex	0.79 ± 0.09	0.61 ± 0.12	0.73 ± 0.06	p=0.292

Table S4. Classification performance of univariate and multivariable clinical models under 2 visits. Values are calculated using bootstrap resampling with N = 1000 repetitions and are presented as mean ± standard deviation. The probability threshold was optimized with respect to balanced accuracy for each model. CDR = Clinical Dementia Rating; MMSE = Mini-Mental State Examination. P-values indicate comparisons between each model and the reference SigLIP with CDR and Age, calculated using independent t-tests, with $p < 0.001$ indicating highly significant differences.

**Variable**	**Text without value**	**Value without textt**	**Text and value**
CDR	Clinical Dementia Rating	0.5	Clinical Dementia Rating: 0.5
MMSE	Mini-Mental State Examination	28	Mini-Mental State Examination: 28
Age	Age	74	Age: 74
Sex	Sex	Female	Sex: Female

Table S5. Examples of encoding strategies for structured clinical variables. The table shows how each variable was represented under the variable label only, observed value only, and variable label plus observed value encoding strategies. In this example, the participant is a 74-year-old female with a CDR score of 0.5 and an MMSE score of 28. CDR = Clinical Dementia Rating; MMSE = Mini-Mental State Examination.

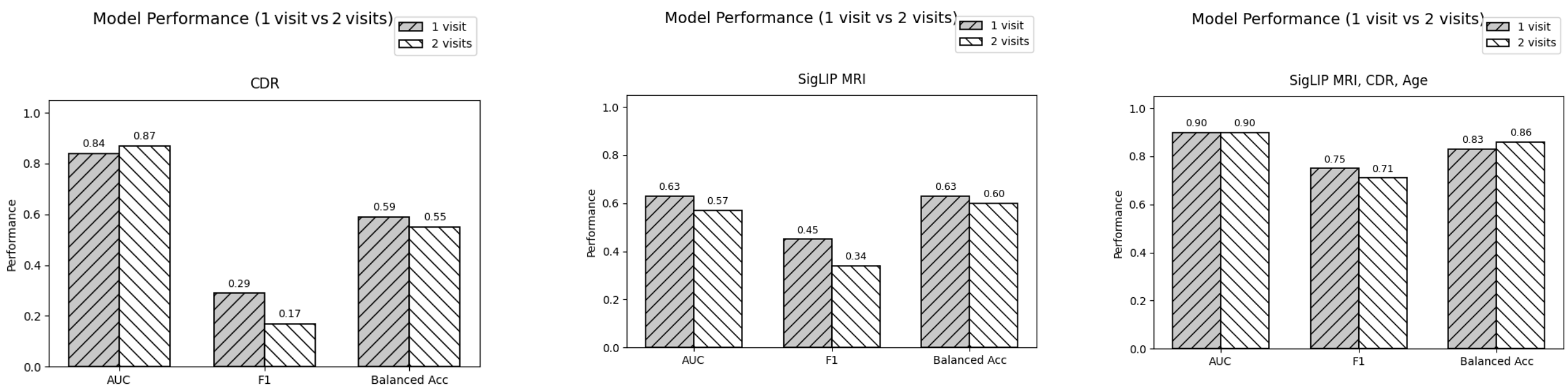

Figure S1.Performance of models using one versus two visits. The models cover a range of unimodal and multimodal configurations, including clinical variable CDR, image-based embeddings (SigLIP MRI), and multimodal combinations (SigLIP MRI with CDR and age).
